# A generalisation study in deep learning-based segmentation of lower-limb muscles across different populations

**DOI:** 10.1038/s41598-026-57242-6

**Published:** 2026-06-08

**Authors:** Zhicheng Lin, Willi Koller, Liruoran Xiong, Haonan Tian, Hans Kainz, Enrico Dall’Ara, Lingzhong Guo

**Affiliations:** 1 School of Electrical and Electronic Engineering, University of Sheffield, Sheffield, UK; 2 Institute of Artificial Intelligence Research, Jiangsu Second Normal University, Nanjing, China; 3 Centre for Sport Science and University Sports, University of Vienna (UniVie), Vienna, Austria; 4 Vienna Doctoral School of Pharmaceutical, Nutritional and Sport Sciences, UniVie, Vienna, Austria; 5 Division of Clinical Medicine, University of Sheffield, Sheffield, UK; 6 Insigneo Institute, University of Sheffield, Sheffield, UK

**Keywords:** Deep learning, Lower-limb muscles, Automatic segmentation, Generalisation, Biomedical engineering, Engineering, Medical imaging

## Abstract

Accurate and consistent segmentation of lower-limb muscles across different populations (e.g., children, young adults, and older individuals) remains challenging due to substantial anatomical differences. This study evaluated the performance of deep learning models for the automatic segmentation of lower-limb muscles in typically developed children (TDC). We present a novel investigation into their generalization ability across different cohorts (healthy young people (HY) and post-menopausal women (PMW)). Our focus was on the Attention-Feature-Fusion-Unet (AFFU) model, which incorporates a feature fusion module into U-Net. First, manual segmentation of T1-weighted images from TDC cohort was conducted by different operators and a reproducibility analysis was evaluated. Then a comparison study was carried out with UNet, UNet +  + , and Attention UNet. The model AFFU achieved the best Dice Similarity Coefficient 0.86 and Relative Volume Error 0.09 on children cohort. It also significantly reduced the Hausdorff Distance and Average Symmetric Surface Distance by approximately 34% and 20%, respectively, compared to the baseline U-Net (*p* < 0.01). It can be observed that larger, regularly shaped muscles achieved higher segmentation accuracy, while smaller and irregular muscles posed difficulties. The experiment showed that a single type of cohort model training is not enough to improve the model generalisation ability. The best results in terms of generalisation were achieved with a training set of mixed multi-class cohorts and a complex model using attention mechanisms.

**Supplementary Information:**

The online version contains supplementary material available at 10.1038/s41598-026-57242-6.

## Introduction

Accurate muscle segmentation in medical imaging is critical for understanding musculoskeletal health, diagnosing diseases, and planning treatments. It can assist clinicians to quantify muscle morphology, monitor volume changes over time, and detect abnormalities associated with various conditions. For instance, some diseases like cerebral palsy, sarcopenia and neurological disorders can cause abnormal muscle growth with muscle loss or motion function abnormity where accurate segmented label could aid clinicians in monitoring the progression of the disease during early diagnosis^1–3^. Despite its importance, muscle segmentation remains a challenging task, particularly in populations with different physiological features, such as children. Compared to adults, children exhibit smaller muscle sizes^4^, greater variability in muscle morphology due to rapid growth, and less-defined muscle boundaries^5^, making precise segmentation even more challenging. The less defined muscle boundaries in children may result from several developmental and physiological factors^5–7^. Children typically have lower intramuscular fat and less developed connective tissue, leading to reduced contrast between muscle and surrounding tissues. In addition, smaller muscle size and lower muscle echo intensity (EI) can further reduce boundary clarity. Ongoing muscle development may also contribute to this effect. As children grow, these factors evolve, leading to more distinct muscle boundaries in adulthood.

Deep learning (DL) models have shown significant advances in automatic medical image segmentation in recent years, demonstrating improved accuracy and efficiency across various clinical applications^8^.. Recent advances in DL have also demonstrated strong potential in disease early diagnosis and clinical decision support, further highlighting the importance of robust and generalisable models in medical imaging applications^9^. DL models rely on manual segmentation results which refers to ground truth (GT). Reproducibility analysis can offer more reliable reference points for assessing the performance of DL models across specific groups^10^. Some muscles have been found to be more reproducible than others for manual segmentation and this needs to be considered when using this information as GT^10^. In previous studies, DL models have been applied in muscle segmentation with good scores, e.g. Ni et al.^11^ designed a cascaded DCNN (deep convolutional neural network) framework to operate automatic segmentation on athletes cohort with Dice Similarity Coefficient (DSC) around 0.90 on 35 lower-limb muscles from 64 subjects (athletes) and Lin et al.^3^ proposed an Attention-Feature-Fusion-UNet (AFFU) and achieved 0.83 DSC on 16 lower-limb muscles on 29 subjects (post-menopausal women). Only a few automatic muscle segmentation studies have been done on children cohort^1^, e.g. Zhu et al.^1^ applied a DL model to segment the lower limb muscles in children (includes 14 male, 6 female with mean age: 9.0 ± 3.2 years old, height: 133.2 ± 22.1 cm, weight: 32.3 ± 16.5 kg.), including 6 of those with cerebral palsy, achieving an average DSC of 0.88.

While previous studies have demonstrated the potential of DL models for muscle automatic segmentation, few have focused on model’s generalisation performance analysis from different age groups which limits the understanding of model reliability and generalisation capability across diverse cohorts. Robust generalisation allows models to be applied across different age cohorts, imaging devices and patients under various condition^12^, making them more clinically viable. Moreover, models with good generalization ability could reduce the cost for retraining of fine-tuning on new dataset^13^ when the new data comes from different or other unseen populations, the well-trained models can be directly used without retraining or fine-tuning of parameters.

However, a critical limitation in most existing studies: they are typically developed and validated on a single, homogeneous population (e.g., healthy young athletes or older women). This severely limits the practical utility of DL models, as a model trained on one population often experiences significant performance degradation when applied to others with vastly different anatomy, tissue contrast, and morphology (e.g., children). The lack of systematic evaluation of model generalization across populations leaves a considerable gap in our understanding of their reliability in real-world clinical settings.

Therefore, this study aimed to address these challenges and gaps through two objectives: 1. Evaluate inter- and intra-operator reproducibility in manual segmentation within a typically developed children (TDC) cohort and establish the reliability of ground truth data, which is foundational for model training and performance evaluation^10,14^. 2. Investigate the generalisation capabilities of the AFFU model^3^ and other popular benchmarks (U-Net^15^, UNet +  +^16^, and Attention U-Net^17^) across datasets spanning children, young adults, and older individuals. By comparing performance across diverse cohorts, this study will uncover potential limitations related to overfitting, underfitting, or cohort-specific biases and better understand domain shift effects and the robustness of segmentation models under heterogeneous conditions by evaluating models on multiple cohorts with distinct anatomical characteristics and analysing both single-cohort and mixed-cohort training strategies. Although the present study focuses on healthy populations, accurate muscle segmentation in such subjects is essential for many clinical and biomechanical applications. For example, subject-specific muscle geometries derived from medical images are widely used in musculoskeletal modelling and finite element (FE) simulations to study joint mechanics, muscle forces, and disease-related changes. Therefore, reliable segmentation in healthy cohorts provides an important reference for both clinical assessment and computational modelling.

## Materials and methods

### Participants

The subjects included 9 typical developed children (TDC), 8 healthy young adults (HY), and 11 healthy post-menopausal women (PMW) (detailed statistical information seen in Table 1).

**Table 1:** Statistical information.

Cohort	Age (year)	Weight (kg)	Height (cm)	BMI
TDC (N = 9) (4F/5 M)	12.1 ± 0.7 (10.8 to 13.0)	42.9 ± 9.6 (34.2 to 62.3)	158 ± 9 (142 to 168)	17.1 ± 2.9 (14.9 to 23.2)
HY (N = 8) (4F/4 M)	26.4 ± 3.5 (22.0 to 31.0)	63.1 ± 8.4 (53.0 to 76.0)	171 ± 10 (160 to 188)	21.4 ± 1.4 (19.0 to 23.9)
PMW (N = 11) (11F)	69.0 ± 6.7 (59.1 to 83.0)	66.9 ± 7.7 (56.8 to 78.6)	159 ± 3 (154 to 164)	26.5 ± 3.4 (21.2 to 31.1)

In the table, four parameters for each cohort are presented as mean ± SD (range: min to max). The number of subjects considered in each cohort, along with gender distribution, is indicated as N = x and xF/yM (females/males).

The T1-weighted TDC images were collected using a Siemens Magnetom Vida Machine (Siemens, Erlangen Germany), with an echo time of 2.46 ms, repetition time of 5.44 ms, flip angle of 9 degrees. The data was collected as part of a larger project^18,19^ with an ethics approval from the ethics committee of University of Vienna (reference number 00578). All scans were acquired with the Dixon method and reconstructed as a composition (voxel size 1.00 × 1.00x1.00 mm^3^, slice spacing 1.00 mm) of 3 single scans from coronal plane. The three reconstructed regions included: hip and proximal femur; middle and distal femur, knee and proximal tibia; tibia and foot.

The PMW cohort were scanned with T1-weighted magnetic resonance images (MR images) and included 11 PMW with no muscle disease who were recruited by the Metabolic Bone Centre (Sheffield, UK) as part of larger studies (approved by the East of England–Cambridgeshire and Hertfordshire Research Ethics Committee and the Health Research Authority, Reference 16/EE/0049)^10^. All the full lower-limb MRI scans were performed on the 1.5T Siemens Magnetom Avanto or 1.5T Siemens Magnetom Aera (Siemens AG, Erlangen, Germany). Four sequences were taken to capture the hip, thigh, knee, and shank^20^. To minimize scanning time without losing detailed joint geometries the joints were scanned with a higher resolution (pixel size of 1.05 × 1.05 mm^2^ and slice thickness of 3.00 mm) than the central portions of the long bones (pixel size of 1.15 × 1.15 mm^2^ and slice thickness of 5.00 mm). The sequences were then combined in MATLAB (R2006a) to create a continuous 3D image from the hip to the ankle ^21^. This was done by standardizing the voxel size of each imaging sequence from the different sections through tri-linear interpolation (interp3, MATLAB R2006a) to be 1.00 × 1.00 × 1.00 mm^3^.

The HY cohort was collected and anonymized as part of the MultiSim project (project title: ‘Clinical data to inform the MultiSim Project: Development of a modeling framework focused on the human musculoskeletal system’, STH Project Number: STH19009, REC Number: 16/EE/0049). The scanning machines and protocol were the same as those used in the PMW cohort.

All methods were carried out in accordance with relevant guidelines and regulations by the Metabolic Bone Centre (Sheffield, UK) and the ethics committee of University of Vienna. And all experimental protocols were approved by University of Sheffield and University of Vienna. Informed consent was obtained from all subjects.

### Deep learning models

Four Convolutional Neural Networks (CNNs) were used in our experimental study: U-Net^15^, UNet +  +^16^, Attention UNet^17^, and AFFU (Attention-Feature-Fusion-UNet)^3^. The first two network architectures are well-known, state-of-the-art designs that have been widely used in the field of medical image segmentation tasks. The AFFU is the modified U-Net structure proposed in^3^ and showed better performance than other benchmark models (U-Net and UNet + +) on the PMW cohort. Although Attention-UNet has not been widely tested in the context of muscle segmentation tasks, it was included in this study as a benchmark model due to its relatively simple attention mechanism compared to AFFU and with lower training costs.

The AFFU is a 2D convolutional neural network designed for medical image segmentation^3^. The model is based on a standard U-Net backbone but incorporates two key modifications. First, it integrates an attention gate mechanism at skip connections, which enhances the focus on anatomically relevant features and suppresses irrelevant background information. Second, it introduces a feature fusion module at the decoder stage, where feature maps from different resolution levels are adaptively weighted and combined. This fusion helps capture both global context and local detail, improving boundary delineation of irregularly shaped muscles. The model operates on individual 2D MR slices, and the 3D muscle volumes are subsequently reconstructed by stacking the segmented slices. The overall architecture is illustrated in Supplementary Figure S1, where the positions of the attention gates and feature fusion blocks are highlighted.

All models were trained using identical protocols on an NVIDIA GeForce RTX™ 3060 Ti GPU with 8 GB of memory. During the training, the input images were randomly cropped to a size of 128 × 128 pixels before being fed into the models which not only reduces computational cost but also helps reduce the impact of image noise. For the model: U-Net, UNet +  + , and Attention-UNet, the batch size was set to 16, while for AFFU, the batch size was set to 8 (due to memory size limitation). The epoch was set to 100 or 200 depending on the application (details given in Section "Deep learning experimental design"). The initial learning rate was 0.001 and multiplied by 0.9 after every 5 epochs. The other hyper-parameters were empirically tuned for best performance^3,4^.

### Data pre-processing

All MR image slices were converted from DICOM (Digital Imaging and Communications in Medicine) to PNG (Portable Network Graphic) format in MATLAB (R2022a). The slices were adjusted into left and right parts according to the body’s symmetry and cropped to a cross section size of 256 × 256 pixels. The MR slices had a resolution of 1.00 × 1.00 mm^2^, with 1.00 mm between slices. The corresponding segmentation labels for each MR image slice were processed using the same protocol.

### Manual segmentation and operator variability analysis of TDC cohort

**Fig. 1:**
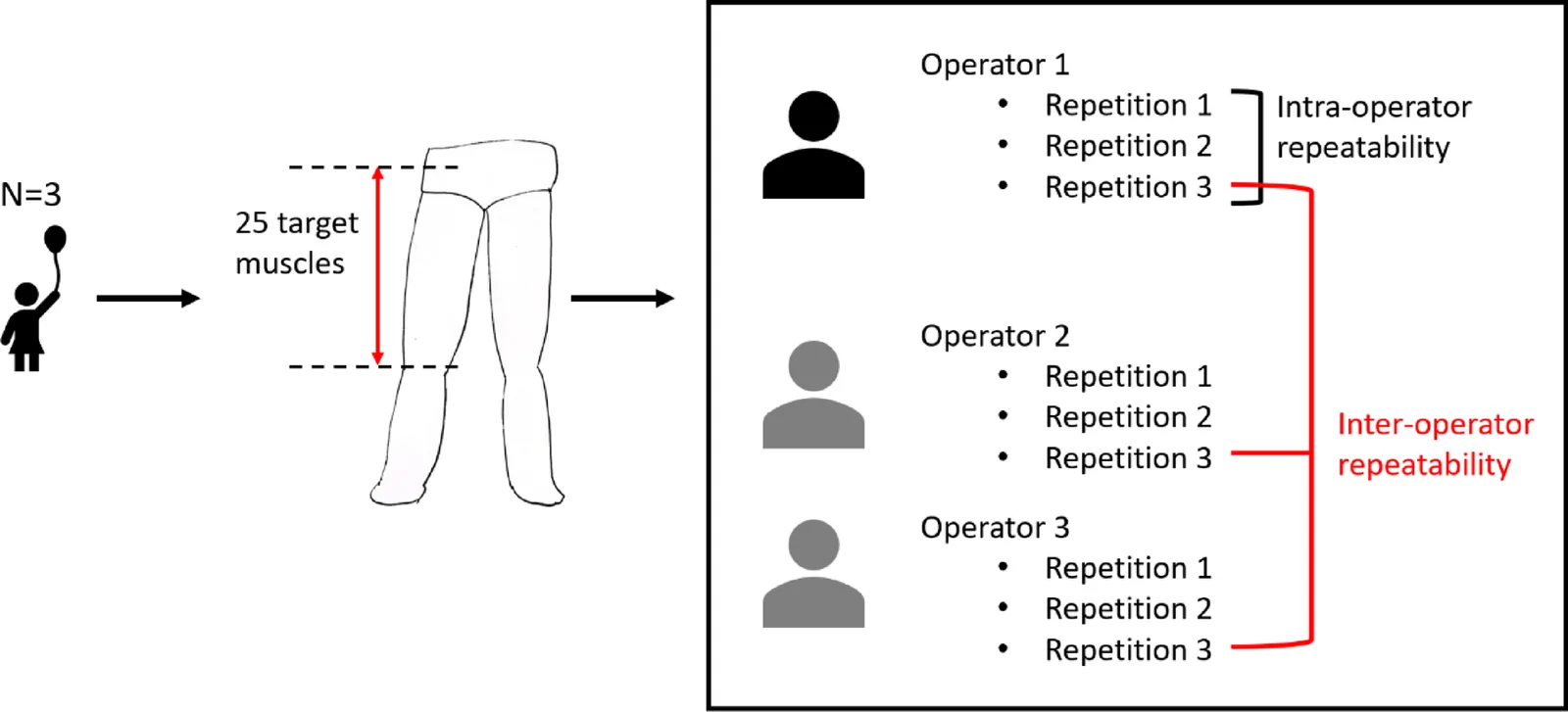
Overview of the manual segmentation operation.

The overview of the methods used in this section was presented in Fig. 1. The MR images of TDC were segmented by three operators: one operator experienced in manual muscle segmentation (ZL), and two operators (LX, HT) who had no previous experience of muscle segmentation. The less experienced operators were trained on at least 15 subjects using Armira (version 2022.2; Thermofisher) to get used to the image segmentation process and the muscle anatomy. In each right limb, 25 muscles of the thigh, from knee to hip, were segmented (Table 3). Three subjects were randomly selected for the reproducibility study. Each operator performed three independent segmentations on the right thigh part of each selected subject. To avoid the influence of previous segmentations, the three segmentations on the same subject were not performed consecutively. The muscle segmentation on both sides of the thigh part of all 9 subjects was performed by the same operator (ZL) and used as the ground truth in further model training.

Inter-operator variability was accessed by calculating the precision error (PE)^14^ associated to the segmentations from the three operators in terms of the muscle volume ($V_{m}$) and length ($L_{m}$). Considering that operators may become familiar with the same case with repeated process, this analysis was based on the third repetition from each operator on each subject. Intra-operator variability was determined by calculating the average PE of the three segmentation results performed by the same operator (ZL) on the three subjects. 10% reproducibility was considered acceptable for the application^12^. Moreover, a specific label was considered to be segmented reproducibly, and thus suitable for subsequent deep learning model training, only when both the PE of $V_{m}$ and $L_{m}$ were lower than 10%. The Psoas muscle was removed from the analysis since in some subjects it was not fully scanned and partially cut off from the view^4,10^.

The pixel resolution of the MR images was 1.00 × 1.00x1.00 mm^3^ so the $V_{m}$ for each muscle was calculated by summing the number of pixels in the labeled segmentation image (MATLAB, R2022a). $L_{m}$ was calculated as the length of the centreline from each muscles’ 3D segmentation results. The centerline was generated by linking the points representing the topological skeleton of each muscle cross-section in the 2D MR images slices^10^. The measurement of $L_{m}$ was automatically performed using the module ‘Vascular Modelling Toolkit—Extract Centerline’ in 3D slicer (version: 5.7.0)^22^.

### Deep learning experimental design

The summary of how the data used in each task can be seen in Table 2 below. Only muscles that showed acceptable reproducibility in the manual segmentation analysis (PE < 10%) were included in the deep learning evaluation tasks. Muscles with high inter- or intra-operator variability were excluded from the quantitative performance analysis to avoid the influence of unreliable ground truth labels on model evaluation. The PE analysis was performed to assess the reproducibility of manual segmentation rather than to restrict the training data. Only muscles with acceptable reproducibility were included in the quantitative evaluation of the deep learning models, in order to ensure that the reported performance reflects model accuracy rather than variability in manual annotation.

**Table 2:** Overview of tasks. The table presents the data cohorts used for training and testing in each task. ‘Number of evaluated muscles’ indicates the number of muscles with highly reproducible segmentation that overlap across corresponding cohorts.

	Training data	Testing data	Number of evaluated muscles
Task 1	TDC	TDC	18
Task 2-a	PMW	TDC	18
Task 2-b	TDC	HY + PMW	16
Task 2-c	TDC + HY + PMW	TDC + HY + PMW	15

#### Task 1: Leave-one-out cross-validation on TDC

Task 1 focused on evaluating the performance of different CNNs (U-Net, UNet +  + , AFFU and Attention-UNet) on TDC images. In this part, models were trained and tested exclusively on MR images from right-side muscles of TDC cohort. During training, a leave-one-out (LOO) cross-validation strategy was employed. In each of the 9 repetitions, data from a single subject (approximately 11% of the total data) was held out as the test set. The data from the remaining 8 subjects (89%) were used for training. 15% of the slices from each subject were randomly selected to form a validation set for hyperparameter tuning and early stopping during each training session. The final model results were averaged across test results from all repetitions.

#### Task 2: Generalisation evaluation across different age groups

Task 2-a: It consisted in testing different CNNs (U-Net and AFFU) on the TDC cohort, which were pre-trained based on the PMW cohort as described in previous study^3^. The objective was to investigate the generalisation performance of the models when trained on a single cohort (PMW) and then directly tested on another cohort (TDC). Additionally, this task aims to compare the performance of AFFU, which integrates additional attention mechanisms, with U-Net to assess whether these attention modules enhance the predictive accuracy of the models, even when trained on a different cohort. The test dataset included the right sides of the thigh muscles from the TDC subjects. Models were trained on the entire PMW cohort (N = 11 subjects). A fixed 20% of the training slices were randomly held out as a validation set for parameter tuning. To reduce the uncertainty in the performance estimates^23^, each model’s training procedure was repeated three times, and the average segmentation results for each target muscle were used to represent the final performance.

Task 2-b: The objective of this task was to test the generalisation performance of the model (U-Net and AFFU) when trained on TDC and then tested on PMW and HY cohorts. Three model weights were randomly chosen from the LOO training in Task 1, and the mean value of each metric on individual muscles was used in the evaluation. In detail, as described in Task 1, for each of the 9 trained models (each trained on 8 TDC subjects, with a validation set from those 8), their weights were saved. Three of these 9 pre-trained models were then randomly selected and directly applied to the entire PMW (N = 11) and HY (N = 8) cohorts for testing, without any further fine-tuning.

Task 2-c: Four models (AFFU, U-Net, UNet +  + and Attention UNet) were trained and tested on subjects from different age groups, including TDC, HY and PMW. The objective was to determine whether the models’ overall feature learning capability remains consistent when trained on different datasets, including multiple age groups, or on a single cohort. In training, each model’s training procedure was repeated three times to reduce the effect of model’s uncertainty, with the average performance on the test dataset presented as the result. For each of the three repetitions, a stratified random sampling was performed to ensure each cohort was represented in all sets. Two subjects were randomly selected from each cohort (TDC, HY, PMW) to form the test set (total 6 subjects). Similarly, two different subjects from each cohort were held out as a fixed validation set (total 6 subjects). The remaining subjects (TDC: 5, HY: 4, PMW: 7; total 16 subjects) were used for training. This ensured that the training, validation, and test sets all contained data from all three populations.

### Evaluation metrics and statistics

For operator variability analysis, the precision error (PE)^14^ was used to assess the reproducibility in segmenting each lower-limb muscle. PE was defined as:

1 $$\begin{array}{*{20}c} {PE = \sqrt {\mathop \sum \limits_{j = 1}^{m} \frac{{CV_{j}^{2} }}{m}} , CV = \frac{SD}{\mu }} \\ \end{array}$$

here CV is coefficient of variance and is defined as the ratio between the standard deviation (SD) and the mean (*μ*) of the properties ($V_{m}$. or $L_{m}$. ) of each muscle, *m*. is the number of subjects (equal to 3 in this study).

For segmentation evaluation, four metrics were used: Dice Similarity Coefficient (DSC), Relative Volume Error (RVE), Hausdorff distance (HD) and Average Symmetric Surface Distance (ASSD)^1,11,20,24^. The selection of evaluation metrics was based on their widespread use in medical image segmentation and their complementary characteristics. DSC is used to quantify the overlap between the predicted segmentation and the ground truth, providing a measure of overall segmentation accuracy. RVE evaluates the difference in segmented volume, which is particularly relevant for clinical applications where precise volume estimation is required. HD measures the maximum boundary deviation between two surfaces, capturing the worst-case segmentation error, while ASSD provides the average boundary distance, offering a more robust assessment osurface agreement. By combining these metrics, both volumetric accuracy and boundary precision can be comprehensively assessed.

Wn comparing two different models or same model’s performance, the differences were tested for normality using the Kolmogorov–Smirnov test, which indicated that the differences between the paired values were not normally distributed. Consequently, a Wilcoxon signed-rank test was employed^3,4^. The paired valu of each model’s DSC, RVE, HD and ASSD metrics were compared across the four models, with a significanclevel of *a = 0.05*.

## Results

### Operator reproducibility analysis of TDC cohort

In Table 3, inter- and intra-operator PE for $V_{m}$ and $L_{m}$ was calculated by three operators (inter-op) and by one experienced operator (ZL) over three repetitions (intra-op) (Supplementary Table S1) on three random chosen subjects from the TDC cohort. The values of inter-op and intra-op PE lower than 10% were marked in light blue and in orange, respectively. Muscles marked in the left column with a dagger symbol (†) or double dagger (‡) denote those muscles that exhibited high reproducibility in the intra- or inter- operator analysis as reported in reference^10^ on post-menopausal women. That study excluded Gemellus superior, Obturator externus, Obturator internus, Pectineus, Piriformis and Psoas. The statistics of inter-operator PE of all operators are shown in Supplementary Table S2.

**Table 3:** Reproducibility of muscle segmentation.

	Intra-op PE [%]		Inter-op PE [%]	
Muscles	$V_{m}$.	$L_{m}$	$V_{m}$	$L_{m}$
Rectus femoris †‡	2.3	3.5	7.8	2.2
Vastus intermedius †‡	3.8	1.4	10.9	15.7
Vastus lateralis †‡	2.7	2.3	7.6	7.4
Vastus medialis †‡	3.1	1.5	7.3	5.3
Sartorius †	2.5	1.6	18.6	1.1
Semimembranosus †‡	2.8	2.2	13.6	5.0
Semitendinosus †‡	1.9	1.2	9.7	4.2
Gracilis †	4.0	0.6	19.4	2.2
Biceps femoris caput brevis †‡	4.5	3.1	24.1	8.3
Biceps femoris caput longum †‡	3.2	1.7	11.1	3.8
Adductor magnus †‡	2.7	3.9	6.9	6.8
Adductor brevis †	24.4	18.3	32.9	37.9
Adductor longus †	5.5	3.9	12.7	7.0
Gemellus superior	21.6	28.0	36.6	44.6
Gluteus maximus †‡	1.2	1.5	6.9	1.6
Gluteus medius	3.0	3.3	4.0	6.7
Gluteus minimus	16.4	7.6	29.6	8.7
Iliacus †‡	5.2	2.8	9.8	3.5
Obturator externus	9.4	35.1	32.6	38.1
Obturator internus	4.8	9.3	17.5	28.7
Pectineus	5.1	4.7	23.4	8.9
Piriformis	7.0	26.5	51.9	27.5
Psoas	4.1	3.4	15.1	12.5
Quadratus femoris	17.5	27.9	32.3	44.6
Tensor fasciae latae †	5.0	2.2	17.8	5.4
	Intra-op PE < 10%		Inter-op PE < 10%	

† or ‡ denote those muscles that exhibited high reproducibility in the intra- or inter- operator analysis as reported in reference^10^ on post-menopausal women.

**Table 4:** Model comparison for the four metrics for models trained on right leg muscles from TDC cohort.

Model	DSC	RVE	HD (mm)	ASSD (mm)
U-Net	0.861 ± 0.054	0.111 ± 0.169	31.5 ± 46.0	2.0 ± 1.4
UNet + +	**0.868 ± 0.045**	0.117 ± 0.133	**28.5 ± 57.7**	**1.9 ± 1.3**
Attention UNet	**0.866 ± 0.049**	0.119 ± 0.155	32.5 ± 52.1	**1.9 ± 1.6**
AFFU	**0.864 ± 0.048**	**0.096 ± 0.161**	**20.8 ± 32.8**	**1.6 ± 0.9**

In Table 4, the values represent the mean (± standard deviation, SD) averaged across 9 TDC subjects using leave-one-out method per model. A total of 162 (9 × 18) labels were calculated for each value. The best values under each metric are highlighted in bold font.

**Table 5:** Model comparison for the four metrics used by U-Net/AFFU trained on PMW cohort.

Model	DSC	RVE	HD (mm)	ASSD (mm)
U-Net	0.531 ± 0.226	− **0.278 ± 0.394**	116.0 ± 87.9	10.9 ± 8.8
AFFU	**0.559 ± 0.250**	− 0.337 ± 0.363	**76.37 ± 66.6**	**9.2 ± 14.2**

In Table 5, DSC, RVE, HD and ASSD found for 18 muscles with high repeatability were included in the automatic segmentation results. Values in the table are the mean value (± standard deviation, SD) averaged across 9 TDC subjects of 3 repetitions of each model, 162 (9 × 18) labels were calculated for each value. The best value under each metrics is highlighted in bold.

The intra-operator PE was lower than the inter-operator PE (*p < 0.01*) for most of the tested muscles (Table 3), excluding $L_{m}$ of Rectus femoris and Sartorius. This result indicates that a single operator tends to be more consistent in muscle segmentation than multiple operators. This consistency is more pronounced in $V_{m}$ measurements than in $L_{m}$.

Muscles like the Adductor brevis, Piriformis, Gemellus superior, Obturator externus and Quadratus femoris exhibited high PE in both inter- and intra-op analyses, indicating challenges in consistently segmenting these muscles regardless of the operator.

**Fig. 2:**
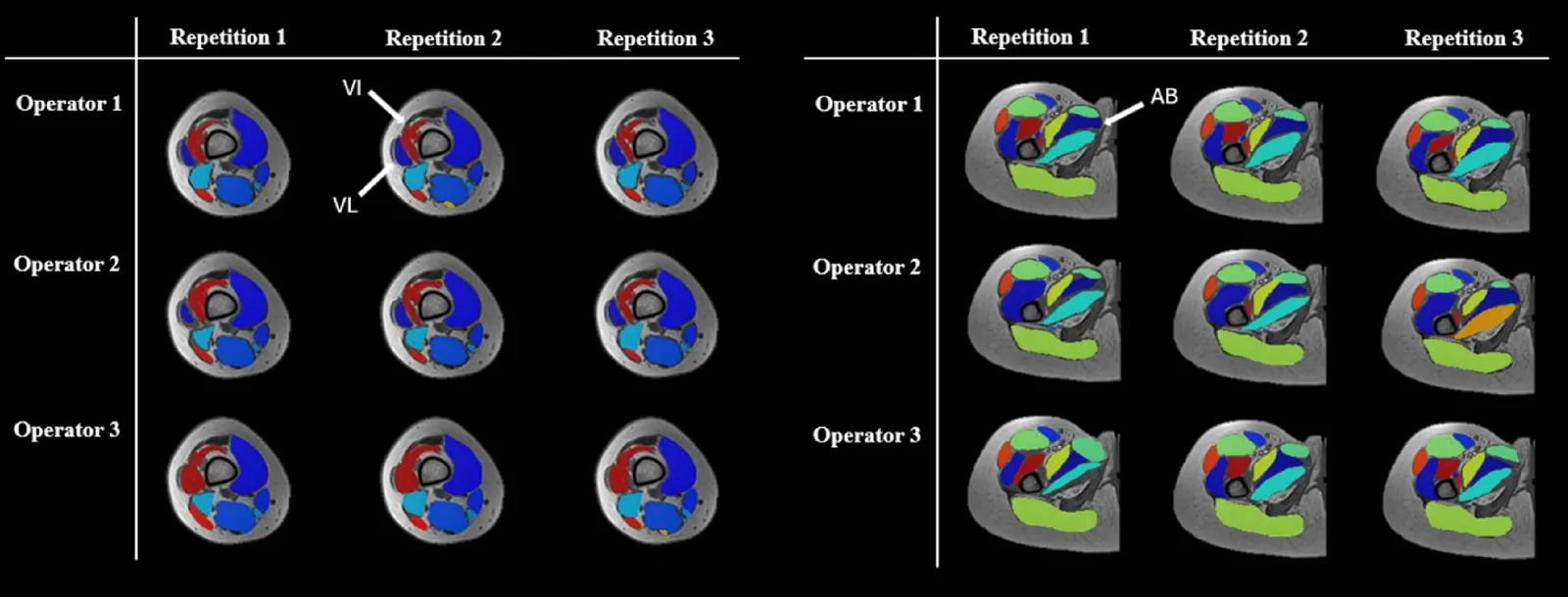
Visualization of manual segmentation across 3 repetitions among 3 operators. The label combined MR images in the upper (right, close to hip part) and lower (left, located at the mid-thigh) sections are from the same subject selected for the repeatability analysis. Repetition 1–3 corresponds to the first through the last trial segments performed by the same operator.

From the manual segmentation visualization (Fig. 2), obvious perceptual differences can be observed in the thigh region (upper section), particularly in muscles like the Vastus intermedius (VI) and Vastus lateralis (VL), especially between operator 3 and the other two operators. Similarly, in areas close to the hips, such as the Adductor brevis (AB), significant differences in boundary delineation between operators are also evident. These differences align with the results shown in Table 3 (the inter-operator PE for the AB exceeds 30%, while the inter-operator PE for the VI is greater than 10%).

Eighteen muscles (e.g. Rectus femoris, Vastus lateralis, and Gluteus maximus) exhibit relatively low PE in intra-op analysis for both $V_{m}$ and $L_{m}$, indicating high reproducibility from the same operator (ZL). These 18 muscles were included in the next DL models’ evaluation stage of Task 1 and Task 2-a. Comparing the intra-operator PE of each operator (Supplementary Table S2), excluding the Biceps femoris caput brevis and Piriformis, the results of individual muscle from different operators show consistency.

Except for $L_{m}$ of the Obturator Internus muscle of subject 1 (CV of 14%), the rest of the muscles marked in blue in Table 3 had a CV lower than 10%.

Among the 16 highly reproducible muscles reported in reference^10^ on 11 subjects and the 18 highly reproducible muscles identified in this section on children, the manual segmentations of 15 muscles are consistently reproducible in both studies and were included in the evaluation stage in Task 2-b/c (where both cohorts were used for training of the models).

In the bilateral muscle parameter comparison (see Supplementary Table S3), the Gluteus maximus, Vastus lateralis, and Adductor magnus exhibited significant volume differences, with the average volume of the right-side muscles being systematically greater than that of the left-side muscles. Other muscles, such as the Rectus femoris and Gracilis, also showed significant differences in either volume or length between the right and left limbs.

### DL model testing results

Task 1 results: TDC test results from model trained on the TDC cohort.

**Fig. 3:**
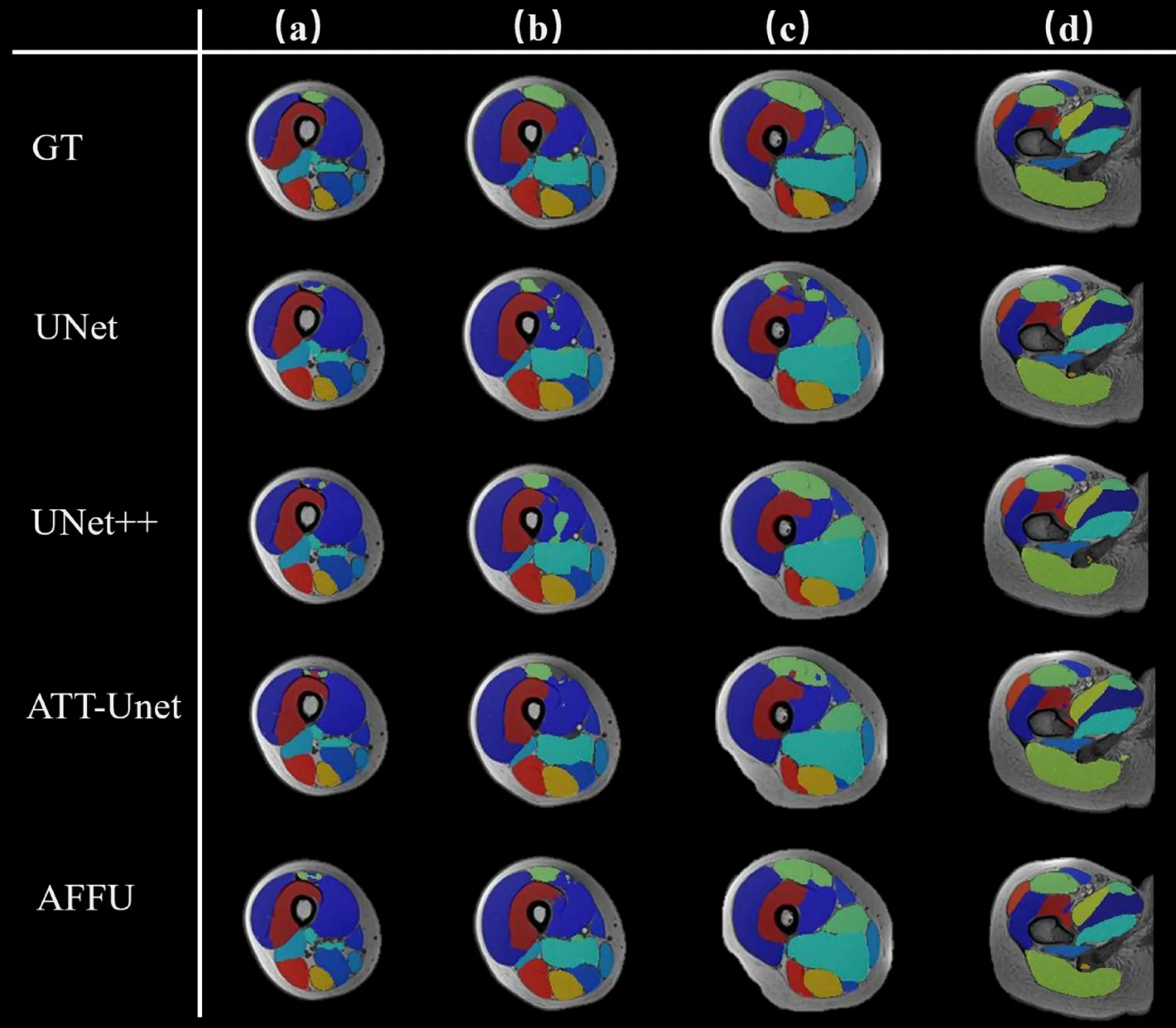
Visualization of model’s predictions in Task 1. The top row represents ground truth (GT). Rows 2–5 represents the predictions from four distinct models. The subject shown in the figure is randomly chosen from test cohorts.

UNet +  + achieved the highest DSC score (0.868 ± 0.045), which is 1% higher than U-Net (0.861 ± 0.054, *p < 0.01*). AFFU achieved the highest score in RVE (0.096 ± 0.161), HD (20.8 ± 32.8 mm) and ASSD (1.6 ± 0.9 mm), outperforming other models with following improvement: 13.5% (U-Net, *p < 0.01*), 17.9% (UNet +  + , *p < 0.01*) and 19.3% (Attention-UNet, *p < 0.01*) in DSC, 34.0% (U-Net, *p < 0.01*), 36.0%(Attention-UNet, *p < 0.01*) in HD, 20% (U-Net, *p < 0.01*) in ASSD. In Fig. 3, it can be seen that compared with others, the basic model UNet shows more mis-segmentation regions and leaves more empty region which should belong to target regions.

Task 2-a results: TDC test results from model trained on the PMW cohort.

When trained on the PMW cohort and tested on TDC cohort, both models didn’t perform well. While the AFFU performed better than U-Net for DSC (0.559, + 5.3%, *p* < 0.05), HD (76.4 mm, − 34.5%, *p* < 0.05) and ASSD (9.2 mm, − 15.6%, *p* < 0.05), the segmentation accuracy is still not accurate enough.

Task 2-b results: PMW/HY test results from model trained on the TDC cohort.

Three random repetitions for each model from the leave-one-out training strategy used in Task 1 were selected and then tested on PMW and HY datasets. Both training and testing were based on the right lower limb muscles, with the evaluation including 16 muscle^3,10^.

Both models failed to segment the muscles from PMW cohort which can be observed from DSC and RVE score (Table 6) for DSC close to 0, RVE close to -1, and showed the similar generalization performance as in Task 2-b on TDC cohort, for DSC around 0.62 and RVE from -0.362 to -0.289.

**Table 6:** Model comparison for the four used metrics of U-Net/AFFU trained on PMW cohort.

	Model	DSC	RVE	HD (mm)	ASSD (mm)
PMW	U-Net	0.060 ± 0.091	− 0.951 ± 0.073	85.2 ± 59.5	28.3 ± 20.5
	AFFU	0.056 ± 0.079	− 0.956 ± 0.069	90.0 ± 57.5	29.6 ± 21.5
HY	U-Net	0.585 ± 0.158	− 0.362 ± 0.245	87.0 ± 57.4	7.5 ± 3.5
	AFFU	0.649 ± 0.133	− 0.289 ± 0.213	74.7 ± 54.7	6.0 ± 2.1

**Table 7:** Model comparison for models trained on combined datasets including different age groups.

Model	DSC	RVE	HD (mm)	ASSD (mm)
U-Net	0.858 ± 0.046	0.145 ± 0.135	**18.4 ± 24.9**	1.7 ± 0.9
UNet + +	**0.870 ± 0.042**	0.143 ± 0.119	**17.5 ± 31.0**	**1.7 ± 1.1**
Attention UNet	**0.869 ± 0.042**	0.149 ± 0.117	**17.8 ± 45.4**	**1.7 ± 1.1**
AFFU	**0.872 ± 0.041**	**0.127 ± 0.117**	**16.7 ± 33.9**	**1.6 ± 1.0**

**Fig. 4:**
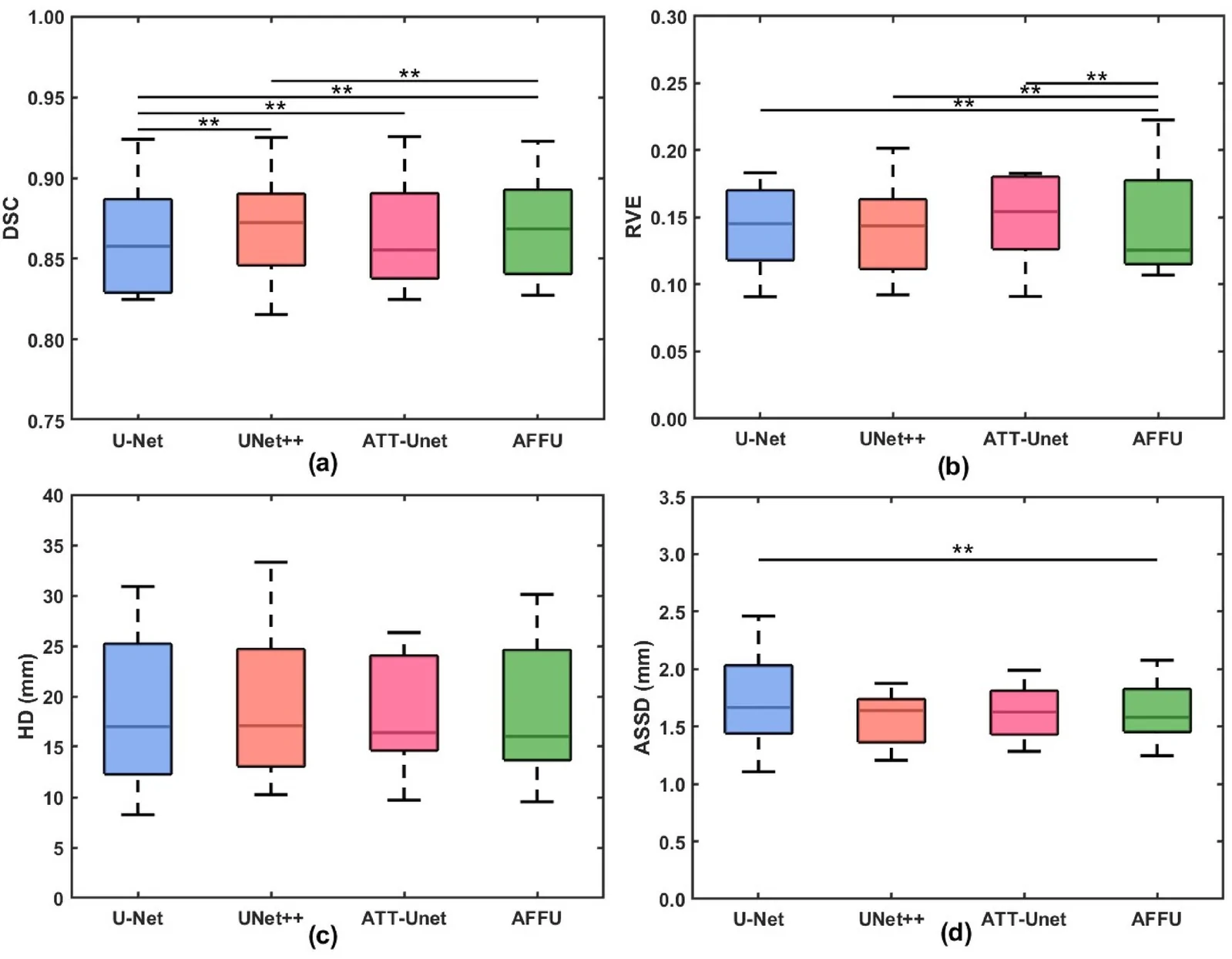
Performance comparison of each model. Box plots of four metrics for U-Net (blue), UNet +  + (orange), Attention-UNet (red) and AFFU (green) are shown (* indicates *p < 0.05*, ** indicates *p < 0.01*). Each boxplot includes 15 medians of each muscle from 9 testing subjects.

**Fig. 5:**
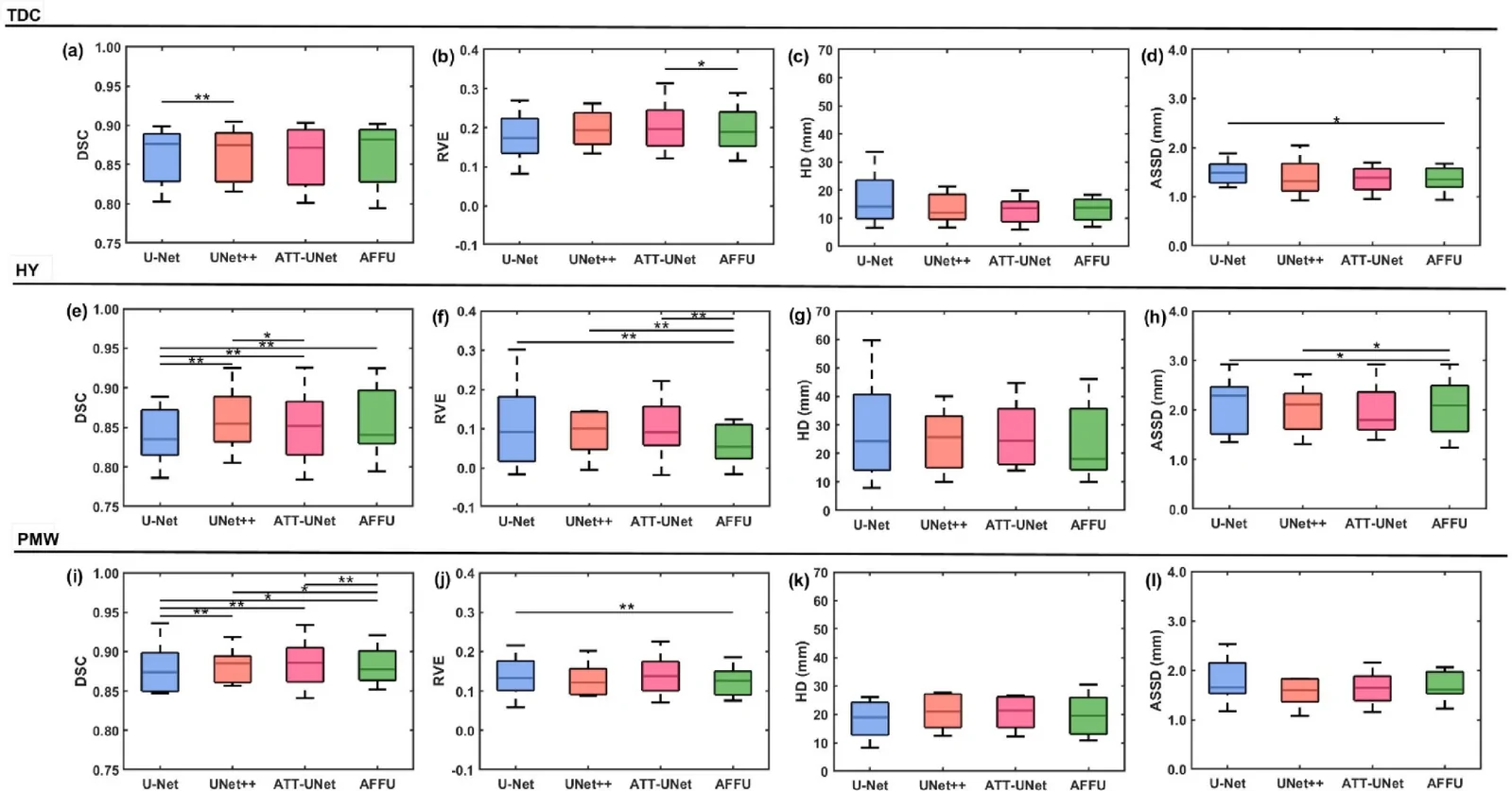
Performance comparison of each model under different cohorts. Box plots of four metrics for U-Net (blue), UNet +  + (orange), Attention-UNet (red) and AFFU (green) are shown (* indicates *p < 0.05*, ** indicates *p < 0.01*). Each boxplot includes 15 medians of each muscle from 3 testing subjects. Panels (**a**–**d**), (**e**–**h**) and (**i**–**l**) represent the results for TDC, YH and PMW, respectively.

**Fig. 6:**
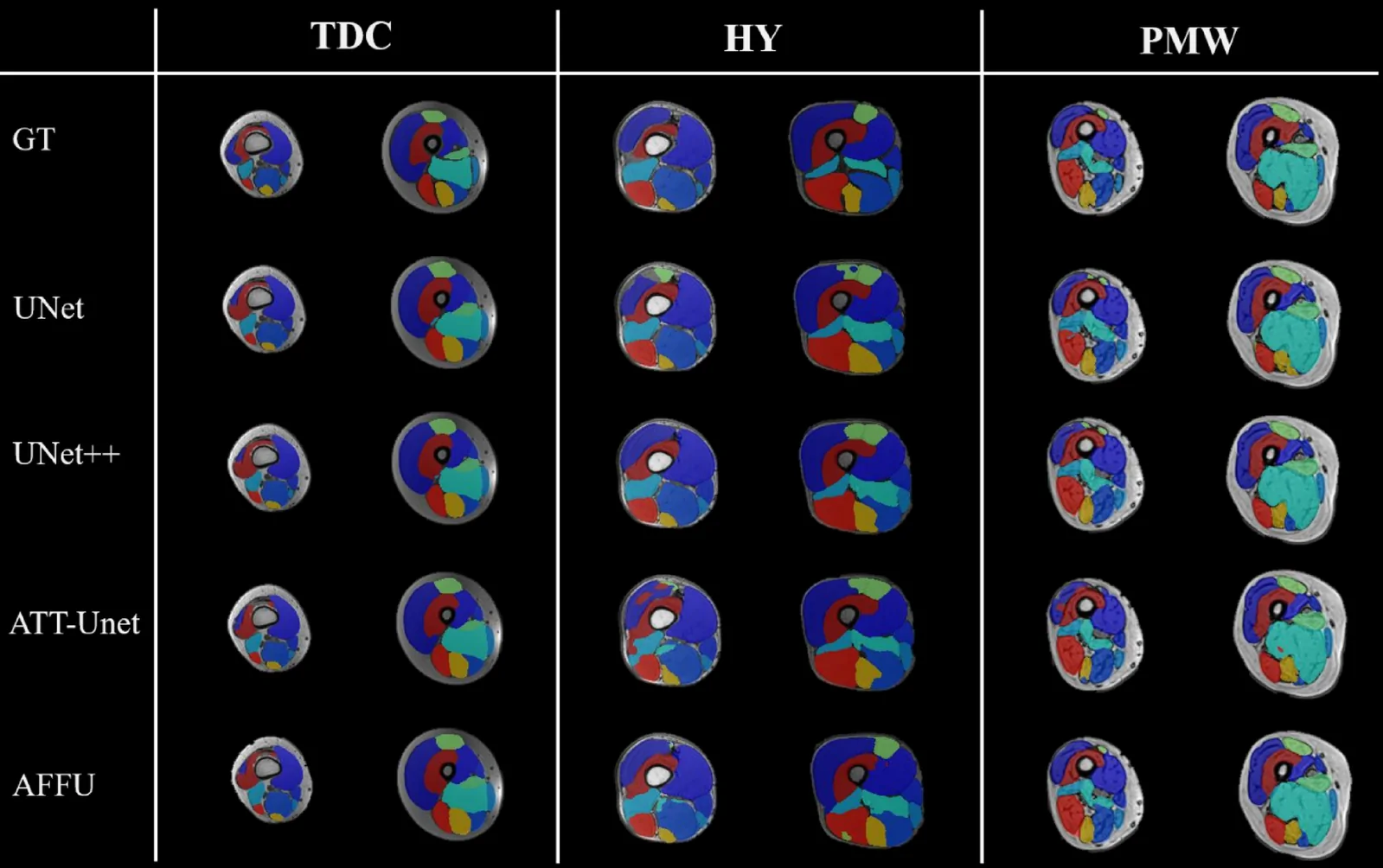
Visualization of model’s predictions. The top row shows the ground truth (GT), while rows 2–5 display predictions from four distinct models. From left to right, columns represent segmentation results for the TDC, HY, and PMW muscle cohorts. Each color corresponds to a specific muscle subregion.

The values in Table 7 represent the mean (± standard deviation, SD) averaged across 9 test subjects from different age groups in 3 repetitions where each repetition includes 1 subject from each cohort. A total of 135 (9 × 15) labels were calculated for each value. The detailed results of each model on each target muscle are shown in Supplementary Table S4. The total performance boxplots are shown in Figs. 4 and 5 and a visualization figure is shown in Fig. 6.

Significant values are in bold.

Task 2-c results: Results from model trained in different age groups.

AFFU provided the best predictions (DSC: 0.872 ± 0.041, RVE: 0.127 ± 0.117, HD: 16.7 ± 33.9 mm, ASSD: 1.6 ± 1.0 mm) among all models, with small significant improvements: 1.6% (U-Net, *p < 0.01*), 0.2% (UNet +  + , *p < 0.01*) in DSC, 12.4% (U-Net, *p < 0.01*), 11.2% (UNet +  + , *p < 0.01*) and 14.8% ( Attention UNet, *p < 0.01*) in RVE, 5.9% (U-Net, *p < 0.01*) in ASSD. All models showed no significant differences in HD (16.7–18.4 mm). For the TDC cohort, AFFU (RVE: 0.187 ± 0.083, ASSD: 1.6 ± 1.1 mm) outperformed Attention UNet (RVE: 0.203 ± 0.010, *p < 0.05*) and U-Net (1.7 ± 0.8 mm, *p < 0.05*) only in terms of RVE and ASSD, respectively, with improvements of 7.9% and 6.7%.

For the HY cohort, AFFU showed better performance on DSC (0.850 ± 0.043), RVE (0.084 ± 0.136) and ASSD (2.1 ± 0.9 mm) compared to others, with following gains: 20.0% (U-Net, *p < 0.01*) in DSC, 31.1% (U-Net, *p < 0.01*), 22.9% (UNet +  + , *p < 0.01*) and 19.2% (Attention UNet, *p < 0.01*) in RVE, 4.5% (U-Net, *p < 0.05*) and 8.7% (UNet +  + , *p < 0.05*) in ASSD. There was no obvious difference in HD between AFFU and other models. For the PMW cohort, AFFU didn’t show better score in HD and ASSD, just outperformed U-Net in DSC and RVE with gain 0.6% (*p < 0.05*) in DSC, 14.6% (*p < 0.01*) in RVE. UNet +  + (DSC: 0.877 ± 0.043) and Attention UNet (0.881 ± 0.034) achieved higher scores in DSC than AFFU (0.874 ± 0.041) with gains 0.3% (*p < 0.05*) and 0.8% (*p < 0.01*), respectively.

During training, the AFFU model is the largest (38M) and takes the longest to train (7.8 min per epoch), while UNet +  + is the smallest (10M) with a slightly longer training time (1.5 min per epoch) compared to U-Net (31M, 1.2 min per epoch) and Attention U-Net (32M, 1.2 min per epoch).

## Discussion

This study aimed to evaluate the generalization ability of different DL models on lower-limb muscles automatic segmentation. In the operator reproducibility analysis (Table 3), 18 muscles achieved high reproducibility scores in the intra-operator assessments, with 15 of these consistent with the results reported by Montefiori et al.^10^ , who characterized 25 thigh muscles in post-menopausal women (PMW) using manual MRI segmentation and found that only a subset of muscles could be reproducibly delineated. Their findings on reproducible muscles are in line with ours, although their cohort was restricted to older women rather than children. Additionally, 8 muscles demonstrated high reproducibility in the inter-operator assessments, with 7 consistent with the results from the PMW cohort^10^. For most of the above-mentioned18 muscles, the coefficients of variation (CV) for muscle volume and length, obtained from the same operator’s segmentations across three different subjects (see Supplementary Table S2), was below 5%. This consistency suggests that the operator’s ability to distinguish most muscles remained reliable, with low variation due to cohort differences, whether in TDC or PMW cohorts. Comparing the intra-operator PE for each operator (see Supplementary Table S2), it can also be seen for most of the muscle, the operators show same repeatability. For the Adductor brevis, Gemellus superior, Obturator externus, Piriformis, and Quadratus femoris muscles, the precision errors (PE) for both volume and length exceeded 10% in both the inter- and intra-operator analyses. This indicates that within the TDC cohort, these muscles were challenging to segment consistently, even by the same operator. As reported in Supplementary Figure S2, Adductor brevis and Obturator externus are surrounded by several other muscles, making their boundaries difficult to define clearly and increasing the likelihood of including portion of the adjacent muscles. The Gemellus superior, Piriformis, and Quadratus femoris, located close to the hip part and relatively small in volume (10–20 cm^3^, see Supplementary Table S3), were more susceptible to regional noise, leading to lower reproducibility. In the bilateral muscle parameter comparison (see Supplementary Table S3), the result was consistent with the findings reported for the PMW cohort^10^. This clearly indicates that caution should be exercised when assuming limb symmetry in the assignment of children’s musculotendon parameters.

The four selected DL models demonstrated good segmentation performance on TDC dataset, achieving an average DSC around 0.86 (Table 4). Overall, the AFFU model, which incorporates feature fusion module, was better because systematically showing the best overall metrics, even if some not significantly different than others. It exhibited the best performance on the RVE metric. Additionally, its results for HD and ASSD were consistent with those reported in^3^, outperforming both the U-Net and UNet +  + . In^1^, the segmentation results for typical developed children achieved a DSC of 0.9 on 13 calf muscles which was higher than in our results. However, our study considered a larger number (N = 18) of target muscles from thigh and hip part. Recent studies on adult cohorts have also reported strong results: Ni et al.^11^ achieved DSC ≈0.90 on 35 muscles in young athletes, while Lin et al.^3^ and Henson et al.^21^ demonstrated that model refinements (e.g., feature fusion, data augmentation) improved segmentation accuracy in post-menopausal women. Yet, all these studies remained restricted to single, homogeneous populations. By contrast, our results highlight that although within-cohort accuracy is comparable, substantial degradation occurs in cross-cohort testing, directly quantifying the domain shift that previous single-cohort studies could not address. This finding is consistent with recent domain generalisation research^13,25,26^, which emphasizes the importance of training on heterogeneous datasets to improve transferability across unseen populations. Several factors may explain this difference: (i) dataset characteristics—calf muscles are more regularly shaped with clearer boundaries on MR images than many hip/thigh muscles, which are larger, irregular, and often surrounded by multiple adjacent structures; (ii) annotation quality—Zhu et al.^1^ reported high-quality, consistent manual segmentations on a relatively small set of calf muscles, whereas our reproducibility analysis showed that some thigh/hip muscles are more challenging even for well-trained operators; (iii) model complexity—Zhu et al. used hybrid 2D/3D networks optimized for calf segmentation, while our work applied a broader benchmark comparison including AFFU, U-Net, UNet +  + , and Attention-UNet across multiple cohorts. In conclusion, these differences suggest that the slightly lower average DSC in our study reflects the greater anatomical and methodological complexity rather than inferior model performance. Among the 18 muscles analyzed, the highest DSC (achieved by AFFU) reached 0.92, with the top five muscles exhibiting DSC values ranging from 0.89 to 0.92. Therefore, the results of our study are also comparable. Furthermore, as shown in Supplementary Table S3 and S4, models achieved higher segmentation performance for large and regularly shaped muscles such as the Rectus Femoris (mean volume:152cm^3^, length: 29.8cm) and Gluteus Maximus (mean volume:432cm^3^, length: 26.0cm), with DSC values over 0.90 and RVE around 0.08. However, for small and irregularly shaped muscles, such as the Obturator Internus (mean volume:22cm^3^, length: 6.4cm), the segmentation performance was comparatively lower. It is likely because large muscles exhibit more distinct boundaries and homogeneous internal texture contrast in MR images, providing more robust and consistent features for the neural network to learn. Conversely, smaller, irregular muscles are more susceptible to partial volume effects and exhibit lower contrast against surrounding tissues, presenting a greater segmentation challenge. In addition, their smaller size increases the impact of partial volume effects and image noise, further contributing to segmentation uncertainty. It should also be noted that direct muscle-wise comparison with existing studies is limited by differences in datasets, anatomical regions, and evaluation protocols. Therefore, the present study focuses on analysing relative performance trends within a consistent experimental setting.

The results from testing models trained on PMW and evaluated on TDC cohort reveal insights into the generalization capabilities of DL models across distinct population groups. As expected, none of the models showed good performance on any of the calculated metrics if trained on a different cohort. Compared to performance on the PMW dataset^3^, both AFFU and U-Net showed worse results on the TDC dataset, with DSC scores decreasing by 32.5% and 34.4%, respectively. When trained on TDC and tested on PMW and HY cohorts, the models also demonstrated unsatisfactory performance (Table 6). Specifically, the worse performance highlights how anatomical differences between the PMW and TDC cohorts may significantly hinder the models’ ability to accurately segment muscle structures. Factors such as variations in muscle size, fat tissue distribution, tissue characteristics and different scanner settings between these groups likely contribute to the decline in segmentation accuracy, emphasizes the importance of training or fine-tuning models on datasets that are representative of the target population^25,26^.

When analysing comprehensive generalisation performance across different cohorts (Fig. 3, Supplementary Table S5)—children (TDC), healthy young people (HY), and post-menopausal women (PMW)—the models exhibit varying degrees of success, and AFFU’s generalisation ability shows robustness. In TDC, AFFU did well in reducing volume error rates and improving surface distance accuracy. Although the overall segmentation accuracy is relatively comparable across models, AFFU’s ability to maintain low error margins indicates better generalisation for this younger age group. For HY people, AFFU demonstrates its most significant improvements in RVE with 0.084 (Fig. 4, Supplementary Table S5). It consistently outperforms other models in all key metrics, indicating that it generalises exceptionally well to this population. The substantial improvement in segmented volume, along with clearer boundary, shows AFFU’s strength in segmenting the often less predictable anatomical features of younger individuals. The results of Task 2-c demonstrate that training on the mixed cohort (TDC, HY, PMW) yielded the most robust model. This is primarily because the mixed dataset encompasses a wider spectrum of anatomical variability (e.g., in muscle size, fat infiltration, morphology), forcing the model to learn the essential features of muscle segmentation rather than memorizing characteristics specific to the training cohort. Recent studies have also emphasized the benefit of incorporating broader anatomical information during training to improve segmentation robustness. For example, in body CT-based muscle segmentation, a “comprehensive muscular consideration” strategy has been shown to improve segmentation performance by learning from multiple muscle regions simultaneously^27^. This is conceptually consistent with our mixed-cohort training strategy, where data from different populations are combined to expose the model to a wider range of anatomical variability. Such diversity encourages the model to learn more generalizable representations of muscle structures, thereby improving its generalisation performance across cohorts. This helps mitigate overfitting to cohort-specific characteristics and improves the model’s ability to generalize across the studied cohorts, although true population-level generalisation remains limited by the number of subjects. For PMW, AFFU’s performance remains competitive, but its advantage is less pronounced compared to other models, particularly in DSC where UNet +  + and Attention-UNet slightly outperform it. However, AFFU still shows better scores in reducing volume errors with 14.5% improvement than others, reinforcing its general ability to provide accurate predictions in more complex cases. This indicates that, while it may not always achieve the highest accuracy for certain age groups, giving different performance in the same cohort reported in other study^5^ where developed ultrasound-based centile reference curves for rectus femoris muscle composition in children aged 4–11 years. While their work focused on non-MRI ultrasound biomarkers rather than deep learning segmentation, it highlights the age-dependent variability in muscle morphology that complicates generalization across cohorts. Under the same conditions, the models combined with attention mechanisms and modules like feature fusion part will show better generalization.

Although the AFFU architecture itself has been described in our previous work, that study was restricted to post-menopausal women and did not investigate model reproducibility or generalisation across different populations. In contrast, the novelty of the present study lies in three main aspects. First, we include three age-diverse cohorts (typically developed children, healthy young adults, and post-menopausal women), enabling a broader evaluation of model performance across heterogeneous populations. Second, we perform a systematic generalisation analysis by explicitly training models on one cohort and testing them on others, thereby quantifying cross-cohort performance degradation and demonstrating the benefit of mixed-cohort training strategies. This provides insight into domain shift effects that have not been sufficiently addressed in previous lower-limb muscle segmentation studies. Third, we conduct a comparative evaluation of AFFU against widely used architectures (U-Net, UNet +  + , and Attention U-Net) under identical experimental settings, allowing us to assess whether more complex architectures incorporating attention and feature fusion offer practical advantages in terms of generalisation. To the best of authors’ knowledge, such a systematic cross-population evaluation has not been thoroughly investigated in this application domain.

This study also has several limitations that could offer directions for future research. First, although the AFFU model excelled in multiple metrics, its higher computational complexity resulted in significantly longer training times compared to other models (e.g., ~ 5 times longer in Task 2-c), which may limit its applicability in resource-constrained environments. Future work could explore model compression techniques, such as knowledge distillation or pruning, as well as more efficient attention mechanisms to improve computational efficiency while maintaining segmentation performance. In addition, in offline analysis, such overhead may be acceptable when higher segmentation accuracy is required. Second, while our quantitative analysis focused on segmentation accuracy, we did not explicitly evaluate the computational cost and parameter efficiency associated with different models. A more comprehensive cost–performance analysis would be valuable for assessing the feasibility of deploying these models in real clinical scenarios. Third, although three distinct cohorts were included in this study, the sample size for each cohort remained relatively small (TDC: N = 9, HY: N = 8, PMW: N = 11), and all images were acquired using similar scanning protocols. While the number of training slices was relatively large, the number of unique subjects remained limited, which may introduce subject-specific biases. Slice-level augmentation improves training stability but cannot fully represent inter-subject variability at the population level. Therefore, the improved performance observed in mixed-cohort training is more likely driven by increased anatomical diversity within the available data rather than true population-level representativeness. In addition, although we designed cross-cohort experiments and mixed-cohort training to evaluate model generalisation, a more detailed sensitivity or subgroup analysis was not performed due to the limited sample size and the high cost of manual segmentation. Such analyses could further quantify the influence of cohort composition, anatomical variability, and acquisition conditions on model performance. Future studies with larger-scale, multi-centre datasets will enable more comprehensive sensitivity and subgroup analyses, providing a more robust evaluation of model generalisation.

## Conclusion

This study investigated the potential of DL based automatic segmentation on children lower-limb muscles and also highlighted the generalisation of advanced models, particularly AFFU, in segmenting muscles across diverse cohorts. When the training is done properly most models perform well. While achieving high accuracy for large and regular muscles, smaller ones remain challenging. Generalisation performance varies significantly across populations, with anatomical differences impacting accuracy. AFFU exhibited robustness but optimization is needed for computational efficiency and population-specific adaptation to enhance broader applicability.

## Supplementary Information

Below is the link to the electronic supplementary material.

Supplementary Material 1

## Data Availability

Data cannot be shared publicly for confidentiality reasons. However, the interested reader can contact the corresponding author to request access to the data. Requests for data may be granted to eligible parties on reasonable requests and with the completion of any required prerequisites, such as a data use agreement. The data of the typically developing children was collected as part of a larger project with an ethics approval from the ethics committee of the University of Vienna (reference number 00578/01,144). Due to confidentiality constraints, the data cannot be shared publicly. However, interested readers may contact hans.kainz@univie.ac.at to request access. Data access may be granted to eligible parties upon reasonable request and the fulfillment of any necessary prerequisites, such as signing a data use agreement. The data of healthy post-menopausal women and young people were recruited by the Metabolic Bone Centre (Sheffield, UK) as part of larger projects (approved by the East of England–Cambridgeshire and Hertfordshire Research Ethics 126 Committee and the Health Research Authority, Reference 16/EE/0049). All data is used anonymously. Data cannot be shared publicly for confidentiality reasons, according to the terms of the Leeds IRB. However, the interested reader can contact info@insigneo.org or the corresponding author to request access to the data. Requests for data may be granted to eligible parties on reasonable requests and with the completion of any required prerequisites, such as a data use agreement.
