# Supplementary material for: A generalisation study in deep learning-based segmentation of lower-limb muscles across different populations
Source: Sci Rep. 2026 Jun 8;16:26136. doi: 10.1038/s41598-026-57242-6 (PMC13490553; doi:10.1038/s41598-026-57242-6)
Supplement: Supplementary file 1 [file 41598_2026_57242_MOESM1_ESM.docx]

Supplementary Table S1 Intra-operator coefficient of variance.

|  | CV of $V_{m}$ (%) | | | CV of $L_{m}$ (%) | | |
| --- | --- | --- | --- | --- | --- | --- |
| Muscles | Sub 1 | Sub 2 | Sub 3 | Sub 1 | Sub 2 | Sub 3 |
| Rectus femoris | 0.7 | 3.3 | 2.1 | 2.8 | 5.2 | 0.8 |
| Vastus intermedius | 2.2 | 4.7 | 4.2 | 0.8 | 1.8 | 1.3 |
| Vastus lateralis | 3.4 | 2.4 | 2.0 | 3.3 | 0.5 | 2.2 |
| Vastus medialis | 2.3 | 3.3 | 3.4 | 2.0 | 0.7 | 1.5 |
| Sartorius | 0.5 | 3.8 | 1.9 | 0.5 | 0.7 | 2.6 |
| Semimembranosus | 0.9 | 4.7 | 0.5 | 3.4 | 1.2 | 0.9 |
| Semitendinosus | 3.1 | 1.0 | 0.4 | 1.4 | 1.4 | 0.7 |
| Gracilis | 2.8 | 4.8 | 4.0 | 0.9 | 0.2 | 0.5 |
| Biceps femoris caput brevis | 5.6 | 5.3 | 1.3 | 3.5 | 3.2 | 2.7 |
| Biceps femoris caput longum | 2.3 | 5.0 | 0.8 | 0.6 | 2.1 | 1.9 |
| Adductor magnus | 4.5 | 0.9 | 1.0 | 4.3 | 5.0 | 1.5 |
| Adductor brevis | 39.6 | 10.1 | 10.4 | 30.0 | 2.2 | 10.0 |
| Adductor longus | 1.8 | 7.9 | 5.1 | 5.7 | 3.1 | 1.8 |
| Gemellus superior | 9.8 | 11.3 | 34.3 | 25.5 | 31.0 | 27.3 |
| Gluteus maximus | 1.3 | 1.3 | 0.9 | 0.2 | 2.5 | 0.5 |
| Gluteus medius | 3.9 | 3.3 | 0.7 | 0.8 | 3.1 | 4.6 |
| Gluteus minimus | 27.4 | 6.0 | 4.5 | 11.2 | 4.7 | 4.9 |
| Iliacus | 2.3 | 7.9 | 4.0 | 3.5 | 2.6 | 2.2 |
| Obturator externus | 14.0 | 7.5 | 3.4 | 5.8 | 37.6 | 62.1 |
| Obturator internus | 6.9 | 3.8 | 2.8 | 14.1 | 4.4 | 6.3 |
| Pectineus | 5.6 | 6.0 | 3.2 | 6.1 | 1.5 | 5.0 |
| Piriformis | 6.9 | 9.0 | 4.0 | 15.5 | 29.0 | 32.2 |
| Psoas | 4.6 | 3.8 | 3.7 | 2.0 | 5.2 | 1.9 |
| Quadratus femoris | 20.0 | 8.1 | 21.2 | 47.5 | 6.5 | 6.9 |
| Tensor fasciae latae | 2.6 | 8.2 | 0.5 | 2.2 | 2.5 | 1.7 |

Intra-operator coefficient of variance (CV) for $V_{m}$ and $L_{m}$ calculated by same operator (ZL) over three repetitions of three subjects (subject 1-3). Muscles marked in blue indicate high reproducibility.

Supplementary Table S2 Intra-operator coefficient of variance.

|  | Intra-op PE of $V_{m}$ [%] | | | Intra-op PE of $L_{m}$ [%] | | |
| --- | --- | --- | --- | --- | --- | --- |
| Muscles | ZL | LX | HT | ZL | LX | HT |
| Rectus femoris | 2.3 | 2.0 | 4.7 | 3.5 | 2.9 | 1.7 |
| Vastus intermedius | 3.8 | 1.7 | 4.9 | 1.4 | 2.6 | 10.1 |
| Vastus lateralis | 2.7 | 4.7 | 4.3 | 2.3 | 2.8 | 1.8 |
| Vastus medialis | 3.1 | 2.6 | 6.3 | 1.5 | 3.1 | 3.8 |
| Sartorius | 2.5 | 2.6 | 8.7 | 1.6 | 1.9 | 1.2 |
| Semimembranosus | 2.8 | 2.3 | 5.5 | 2.2 | 7.9 | 2.6 |
| Semitendinosus | 1.9 | 2.5 | 5.7 | 1.2 | 4.1 | 1.3 |
| Gracilis | 4.0 | 1.9 | 9.1 | 0.6 | 2.1 | 2.1 |
| Biceps femoris caput brevis | 4.5 | 24.6 | 19.5 | 3.1 | 26.9 | 17.0 |
| Biceps femoris caput longum | 3.2 | 5.8 | 4.0 | 1.7 | 2.3 | 3.2 |
| Adductor magnus | 2.7 | 6.6 | 3.7 | 3.9 | 4.8 | 6.1 |
| Adductor brevis | 24.4 | 40.4 | 25.4 | 18.3 | 35.0 | 7.7 |
| Adductor longus | 5.5 | 6.9 | 5.9 | 3.9 | 4.8 | 6.5 |
| Gemellus superior | 21.6 | 41.2 | 12.8 | 28.0 | 32.6 | 38.4 |
| Gluteus maximus | 1.2 | 2.5 | 3.3 | 1.5 | 1.3 | 1.5 |
| Gluteus medius | 3.0 | 38.8 | 6.2 | 3.3 | 43.7 | 3.1 |
| Gluteus minimus | 16.4 | 11.3 | 12.6 | 7.6 | 8.2 | 6.6 |
| Iliacus | 5.2 | 22.3 | 4.7 | 2.8 | 21.0 | 2.0 |
| Obturator externus | 9.4 | 25.9 | 19.5 | 42.1 | 16.0 | 26.9 |
| Obturator internus | 4.8 | 10.7 | 12.3 | 9.3 | 9.0 | 17.6 |
| Pectineus | 5.1 | 9.5 | 9.9 | 4.7 | 6.6 | 4.0 |
| Piriformis | 7.0 | 50.8 | 13.6 | 26.5 | 27.7 | 23.2 |
| Psoas | 4.1 | 11.5 | 5.4 | 3.4 | 7.3 | 3.9 |
| Quadratus femoris | 17.5 | 14.5 | 54.8 | 27.9 | 55.7 | 15.5 |
| Tensor fasciae latae | 5.0 | 3.3 | 3.4 | 2.2 | 4.5 | 5.2 |
|  | Intra-op PE < 10% | | | | | |

Intra-operator PE for $V_{m}$ and $L_{m}$of each operator (ZL: experienced operator, LX/HT: less experienced operator) were shown in the table. The values intra-op PE lower than 10% were marked in orange.

Supplementary Table S3 Muscle volume and length variability analysis.

| Muscles | Volume (cm3) | | | Length (cm) | | |
| --- | --- | --- | --- | --- | --- | --- |
|  | Left | Right | Difference | Left | Right | Difference |
| Rectus femoris | 135 (84, 207) ^**^ | 159 (98, 240) | 18 % | 29.3 (27.8, 38.8) | 30.1 (27.1, 32.9) | 3 % |
| Vastus intermedius | 255 (191, 349) | 246 (188, 331) | -3 % | 33.5 (30.2, 39.5) | 32.3 (29.4, 39.8) | -3 % |
| Vastus lateralis | 358 (265, 403) ^*^ | 410 (294, 453) | 14 % | 32.1 (26.6, 34.5) | 30.7 (27.2, 34.3) | -4 % |
| Vastus medialis | 218 (150, 295) | 227 (164, 296) | 4 % | 30.2 (27.5, 33.5) | 29.9 (25.1, 35.5) | -1 % |
| Sartorius | 62 (54, 82) | 69 (49, 110) | 12 % | 49.0 (42.6, 51.1) | 48.2 (42.6, 50.4) | -2 % |
| Semimembranosus | 100 (90, 151) | 110 (85, 166) | 10 % | 26.7 (24.6, 29.9) | 25.3 (21.2, 30.4) | -5 % |
| Semitendinosus | 94 (61, 120) ^*^ | 107 (68, 130) | 14 % | 31.0 (26.1, 33.8) | 31.4 (24.7, 34.4) | 1 % |
| Gracilis | 45 (31, 54) | 48 (32, 63) | 8 % | 32.5 (24.6, 35.2) ^*^ | 30.6 (25.6, 32.2) | -6 % |
| Biceps femoris caput brevis | 50 (32, 63) | 49 (29, 85) | -2 % | 22.7 (21.4, 26.0) | 23.1 (18.8, 26.5) | 2 % |
| Biceps femoris caput longum | 93 (58, 138) | 101 (52, 146) | 9 % | 27.7 (23.4, 32.8) ^**^ | 25.6 (22.6, 30.7) | -7 % |
| Adductor magnus | 279 (206, 363) ^**^ | 304 (214, 405) | 9 % | 28.5 (22.6, 31.6) | 28.4 (20.7, 29.4) | 0 % |
| Adductor brevis | 61 (37, 70) | 58 (34, 79) | -6 % | 13.7 (12.3, 16.2) | 12.5 (9.6, 14.8) | -8 % |
| Adductor longus | 64 (52, 80) | 74 (47, 97) | 15 % | 19.8 (17.1, 22.7) ^**^ | 18.5 (14.8, 21.8) | -6 % |
| Gemellus superior | 5 (4, 9) | 5 (3, 8) | 3 % | 3.8 (3.1, 6.2) | 4.5 (3.6, 7.9) | 17 % |
| Gluteus maximus | 407 (281, 553) ^**^ | 465 (296, 602) | 14 % | 25.9 (22.8, 28.5) | 26.0 (24.1, 28.4) | 0 % |
| Gluteus medius | 179 (108, 205) | 181 (118, 214) | 2 % | 15.7 (14.7, 18.2) | 15.7 (13.5, 18.5) | 0 % |
| Gluteus minimus | 48 (38, 61) | 51 (38, 70) | 7 % | 12.4 (9.3, 13.7) | 10.2 (4.8, 12.8) | -18 % |
| Iliacus | 97 (68, 113) | 99 (67, 123) | 3 % | 22.7 (19.3, 24.6) | 21.5 (19.5, 23.3) | -5 % |
| Obturator externus | 26 (20, 35) | 24 (17, 30) | -9 % | 6.1 (3.5, 10.4) | 7.8 (6.7, 9.4) | 28 % |
| Obturator internus | 28 (21, 35) ^**^ | 24 (15, 30) | -16 % | 8.3 (7.0, 9.2) ^**^ | 6.6 (5.1, 8.5) | -21 % |
| Pectineus | 31 (23, 36) | 33 (23, 45) | 7 % | 10.6 (9.4, 12.6) | 10.5 (9.5, 12.4) | 0 % |
| Piriformis | 18 (10, 22) | 16 (10, 23) | -11 % | 5.2 (4.2, 8.0) | 6.2 (4.6,7.5) | 20 % |
| Quadratus femoris | 10 (2,14) | 13 (2, 17) | 31 % | 4.4 (3.2, 5.6) | 5.6 (2.8, 6.4) | 27 % |
| Tensor fasciae latae | 32 (19, 39) | 30 (23, 55) | -7 % | 14.2 (12.2, 16.5) | 13.8 (12.2, 15.4) | -2 % |

Median (minimum, maximum) values of muscle (excluding Psoas) volume and length for the right and left lower-limb muscles. Muscles marked in the column Left with * or ** denote significant difference ($p<0.01$or $p<0.05$) between limbs in $V_{m}$ and $L_{m}$, respectively. The difference between is calculated as (Median_R – Median_L)/Median_L. All the segmentation results used were performed by the same operator.

Supplementary Table S4 Model performance under four metrics on TDC cohort.

|  | **DSC** | | | | **RVE** | | | |
| --- | --- | --- | --- | --- | --- | --- | --- | --- |
| **Muscles** | **U-Net** | **UNet++** | **ATT-UNet** | **AFFU** | **U-Net** | **UNet++** | **ATT-UNet** | **AFFU** |
| Rectus femoris | 0.904 | 0.908 | 0.911 | 0.899 | 0.103 | 0.088 | 0.078 | 0.074 |
| Vastus intermedius | 0.870 | 0.867 | 0.868 | 0.875 | 0.092 | 0.117 | 0.098 | 0.095 |
| Vastus lateralis | 0.905 | 0.905 | 0.906 | 0.909 | 0.092 | 0.088 | 0.077 | 0.070 |
| Vastus medialis | 0.897 | 0.902 | 0.894 | 0.896 | 0.167 | 0.163 | 0.169 | 0.153 |
| Sartorius | 0.842 | 0.846 | 0.842 | 0.843 | 0.153 | 0.228 | 0.180 | 0.153 |
| Semimembranosus | 0.887 | 0.884 | 0.886 | 0.891 | 0.142 | 0.149 | 0.137 | 0.108 |
| Semitendinosus | 0.887 | 0.889 | 0.887 | 0.887 | 0.116 | 0.124 | 0.104 | 0.103 |
| Gracilis | 0.853 | 0.846 | 0.848 | 0.858 | 0.185 | 0.224 | 0.189 | 0.168 |
| Biceps femoris caput brevis | 0.852 | 0.845 | 0.864 | 0.864 | 0.161 | 0.134 | 0.135 | 0.098 |
| Biceps femoris caput longum | 0.879 | 0.896 | 0.886 | 0.895 | 0.114 | 0.121 | 0.101 | 0.113 |
| Adductor magnus | 0.893 | 0.894 | 0.892 | 0.895 | 0.128 | 0.122 | 0.125 | 0.139 |
| Adductor longus | 0.850 | 0.852 | 0.847 | 0.846 | 0.190 | 0.162 | 0.124 | 0.138 |
| Gluteus maximus | 0.909 | 0.916 | 0.914 | 0.915 | 0.125 | 0.116 | 0.107 | 0.104 |
| Gluteus medius | 0.850 | 0.865 | 0.865 | 0.856 | 0.064 | 0.110 | 0.051 | 0.034 |
| Iliacus | 0.827 | 0.832 | 0.831 | 0.826 | 0.208 | 0.251 | 0.168 | 0.130 |
| Obturator internus | 0.777 | 0.761 | 0.774 | 0.780 | 0.421 | 0.527 | 0.371 | 0.355 |
| Pectineus | 0.785 | 0.802 | 0.801 | 0.803 | 0.113 | 0.210 | 0.203 | 0.149 |
| Tensor fasciae latae | 0.814 | 0.851 | 0.836 | 0.839 | 0.115 | 0.153 | 0.103 | -0.039 |
|  | **HD** | | | | **ASSD** | | | |
| **Muscles** | **U-Net** | **UNet++** | **ATT-UNet** | **AFFU** | **U-Net** | **UNet++** | **ATT-UNet** | **AFFU** |
| Rectus femoris | 13.629 | 17.903 | 14.900 | 14.824 | 1.181 | 1.242 | 1.157 | 1.121 |
| Vastus intermedius | 37.549 | 17.489 | 36.016 | 13.958 | 2.196 | 1.618 | 2.075 | 1.557 |
| Vastus lateralis | 24.271 | 32.314 | 31.041 | 16.779 | 1.585 | 2.091 | 1.980 | 1.468 |
| Vastus medialis | 56.590 | 37.570 | 39.708 | 30.055 | 2.354 | 1.982 | 2.091 | 2.290 |
| Sartorius | 10.675 | 42.908 | 29.096 | 9.059 | 1.040 | 1.612 | 1.323 | 1.336 |
| Semimembranosus | 27.706 | 29.506 | 35.689 | 18.935 | 1.576 | 1.677 | 1.897 | 1.690 |
| Semitendinosus | 16.427 | 13.998 | 14.801 | 26.542 | 1.282 | 1.227 | 1.226 | 1.246 |
| Gracilis | 12.120 | 12.109 | 15.730 | 37.768 | 1.003 | 1.052 | 1.032 | 0.995 |
| Biceps femoris caput brevis | 65.873 | 55.097 | 49.879 | 53.396 | 2.284 | 2.259 | 1.901 | 1.799 |
| Biceps femoris caput longum | 47.586 | 38.534 | 24.911 | 19.074 | 1.955 | 1.755 | 1.548 | 1.937 |
| Adductor magnus | 36.845 | 27.829 | 26.387 | 23.929 | 2.511 | 2.170 | 2.141 | 2.198 |
| Adductor longus | 17.634 | 42.906 | 39.519 | 40.775 | 1.652 | 2.042 | 2.102 | 1.848 |
| Gluteus maximus | 80.275 | 58.145 | 55.205 | 29.601 | 3.373 | 2.501 | 2.655 | 2.019 |
| Gluteus medius | 17.738 | 14.974 | 50.298 | 18.504 | 1.845 | 1.723 | 2.698 | 1.998 |
| Iliacus | 46.135 | 26.067 | 47.027 | 21.443 | 3.196 | 2.088 | 2.453 | 2.059 |
| Obturator internus | 76.346 | 22.039 | 25.476 | 17.844 | 4.285 | 2.313 | 2.251 | 2.077 |
| Pectineus | 16.714 | 12.126 | 11.780 | 14.890 | 1.960 | 1.667 | 1.657 | 1.732 |
| Tensor fasciae latae | 20.413 | 11.207 | 27.399 | 10.778 | 1.838 | 1.175 | 1.677 | 1.155 |

Each value recorded in the table represents the mean value for all test subjects (9 in total) under the leave-one-out training strategy.

Supplementary Table S5 Model comparison for different age groups.

|  | **Model** | **DSC** | **RVE** | **HD (mm)** | **ASSD (mm)** |
| --- | --- | --- | --- | --- | --- |
| **TDC** | U-Net | 0.860 ± 0.040 | 0.189 ± 0.118 | 22.3 ± 33.3 | 1.7 ± 0.8 |
|  | UNet++ | 0.869 ± 0.035 | 0.190 ± 0.097 | 22.2 ± 30.2 | 1.8 ± 1.3 |
|  | Attention UNet | 0.867 ± 0.037 | 0.203 ± 0.010 | 41.2 ± 72.7 | 1.9 ± 1.7 |
|  | AFFU | 0.866 ± 0.037 | 0.187 ± 0.083 | 29.1 ± 50.0 | 1.6 ± 1.1 |
| **HY** | U-Net | 0.835 ± 0.050 | 0.122 ± 0.167 | 27.8 ± 19.4 | 2.2 ± 0.9 |
|  | UNet++ | 0.851 ± 0.045 | 0.109 ± 0.137 | 34.3 ± 41.3 | 2.3 ± 1.1 |
|  | Attention UNet | 0.845 ± 0.047 | 0.104 ± 0.143 | 28.5 ± 22.0 | 2.1 ± 0.8 |
|  | AFFU | 0.850 ± 0.043 | 0.084 ± 0.136 | 31.3 ± 24.5 | 2.1 ± 0.9 |
| **PMW** | U-Net | 0.869 ± 0.042 | 0.137 ± 0.106 | 25.6 ± 19.6 | 2.0 ± 0.9 |
|  | UNet++ | 0.877 ± 0.043 | 0.131 ± 0.108 | 25.0 ± 15.2 | 1.9 ± 0.8 |
|  | Attention UNet | 0.881 ± 0.034 | 0.131 ± 0.080 | 26.3 ± 19.8 | 1.9 ± 0.7 |
|  | AFFU | 0.874 ± 0.041 | 0.117 ± 0.101 | 24.3 ± 19.3 | 2.0 ± 0.9 |

Supplementary Figure S1 The structure of AFFU. The Attention-Feature-Fusion-UNet (AFFU) was proposed based UNet to explore the employment of multiple attention mechanisms to achieve the desired segmentation accuracy. The AFFU incorporated attention gates into each individual layer and used CBAM block to help compensate for any deficiencies in a single attention gate, and mitigated losses during resizing, enhancing the neural network's receptive field using a pyramid pooling technique.

Supplementary Figure S2 Visualization of manual segmentation on muscles with low reproducibility. 5 muscles were included and in different colour. (AB: Adductor brevis (orange), GS: Gemellus superior (blue), OE: Obturator externus (red), PI: Piriformis (yellow), QF: Quadratus femoris (tiffany blue))
